# Assessment of perineural spread in advanced cutaneous squamous cell carcinomas treated with immunotherapy

**DOI:** 10.1186/s40644-024-00678-8

**Published:** 2024-03-18

**Authors:** Karda Cavanagh, Luke S. McLean, Annette M. Lim, Anthony Cardin, Sidney M. Levy, Danny Rischin

**Affiliations:** 1 Department of Cancer Imaging, Peter MacCallum Cancer Centre, Melbourne, VIC Australia; 2 Department of Medical Oncology, Peter MacCallum Cancer Centre, Melbourne, VIC Australia; 3 Department of Oncology, Sir Peter MacCallum, University of Melbourne, Melbourne, VIC Australia; 4 Department of Nuclear Medicine, Royal Melbourne Hospital, Melbourne, VIC Australia

**Keywords:** Cutaneous squamous cell carcinoma, Immunotherapy, Perineural spread

## Abstract

**Background:**

Cutaneous squamous cell carcinoma (CSCC) has a propensity for perineural spread (PNS) which is associated with poorer treatment outcomes. Immunotherapy is the new standard of care treatment for advanced CSCC resulting in durable responses. PNS is not captured by traditional response assessment criteria used in clinical trials, e.g. RECIST 1.1, and there is limited literature documenting radiological PNS responses to immunotherapy. In this study we assess PNS responses to immunotherapy using a modified grading system.

**Methods:**

This is an Australian single-center retrospective review of patients with advanced CSCC who were treated with immunotherapy between April 2018 and February 2022 who had evidence of PNS on pre-treatment magnetic-resonance imaging (MRI). The primary outcome was blinded overall radiological response in PNS using graded radiological criteria, post-commencement of immunotherapy. Three defined timepoints (< 5 months, 5–10 months, > 10 months) were reviewed. Secondary outcomes included a correlation between RECIST 1.1 and PNS assessments and the assessment of PNS on fluorodeoxyglucose (FDG)-positron emission tomography (PET)/computed tomography (CT).

**Results:**

Twenty CSCC patients treated with immunotherapy were identified. Median age was 75.7 years and 75% (*n* = 15) were male. All patients had locoregionally advanced disease and no distant metastases. Median follow-up was 18.5 months (range: 2–59). 70% (*n* = 14) demonstrated a PNS response by 5 months. Three patients experienced pseudoprogression. One patient had PNS progression by the end of study follow up. RECIST 1.1 and PNS responses were largely concordant at > 10 months (Cohen’s Kappa 0.62). 5/14 cases had features suspicious for PNS on FDG-PET/CT.

**Conclusions:**

PNS response to immunotherapy can be documented on MRI using graded radiological criteria. High response rates were seen in PNS with the use of immunotherapy in this cohort and these responses were largely concordant with RECIST 1.1 assessments. FDG-PET/CT demonstrated limited sensitivity in the detection of PNS.

**Supplementary Information:**

The online version contains supplementary material available at 10.1186/s40644-024-00678-8.

## Background

Keratinocyte cancers, including cutaneous squamous cell carcinoma (CSCC), are the most common cancers in humans with 2–3 million occurrences per year worldwide. CSCC incidence has increased 310% between 1990 to 2017 and most frequently arises in the head and neck [1, 2]. Immunotherapy is now the standard of care for advanced CSCC with the potential for durable clinical and radiological responses and improvements in quality of life [3–7].

Perineural spread (PNS) is the radiological descriptor for the process of macroscopic tumour invasion into named nerves. Whilst PNS is uncommon, it can cause significant morbidity due to frequent involvement of the head and neck region by CSCC [8]**.** Historically PNS has been associated with poorer treatment outcomes [9, 10]. There has been a lack of consensus on the management of locally advanced CSCC with perineural involvement with treatment consisting of aggressive surgical resections or radiotherapy with or without chemotherapy [11–13]. With the use of immunotherapy in advanced CSCC, response rates of around 50% are reported [3–6] and impressive major pathological response rates of 50% have also been seen in the neoadjuvant setting with the use of cemiplimab [14]. However, there is only limited retrospective data reporting on PNS responses with immunotherapy and there is no consensus on how to best assess this [15].

There are no prospectively validated approaches for assessing PNS response to immunotherapy and PNS is not incorporated into validated tumour response assessment criteria such as RECIST 1.1 [16]. In CSCC, the requirement for RECIST 1.1 measurable disease in clinical trials has meant that patients with perineural disease only have been excluded from the key immunotherapy studies to date as PNS is often detected as thickening of a nerve with enhancement without a RECIST measurable component. Understanding if PNS responses to immunotherapy can be seen on conventional imaging techniques such as MRI or FDG-PET/CT is of relevance in CSCC in clarifying disease status, overall treatment response and in aiding choice of surveillance imaging modality for PNS predominant disease. Additionally, the use of RECIST 1.1 in the setting of immunotherapy is limited by its inability to capture pseudoprogression. Whilst newer criteria such as irRECIST have been developed in an attempt to capture immunotherapy-related treatment concepts including pseudoprogression, these criteria still fail to capture changes in PNS [17]. MRI scanning represents an excellent tool to assess PNS due to superior soft tissue contrast and multiplanar imaging allowing assessment of the skull base [18–20]. Contrast enhancement on MRI reflects a disrupted blood-nerve barrier in PNS and fat suppression may be used to enhance assessment of tumour infiltrated nerves [21–23].

This study aims to describe PNS responses in a cohort of patients with advanced CSCC and PNS on baseline magnetic resonance imaging (MRI) who were treated with immunotherapy, using modified graded radiological criteria. In addition, we correlate PNS assessments with RECIST 1.1 response and review the utility of identifying PNS on fluorodeoxyglucose (FDG)-positron emission tomography (PET)/computed-tomography (CT).

## Methods

This study was a single-centre retrospective review of patients with advanced CSCC who had PNS identified on baseline MRI prior to the commencement of immunotherapy. Medical records between April 2018 and February 2022 were reviewed to identify cases. For inclusion, patients must have had advanced CSCC defined as either metastatic or locally advanced disease not amenable to curative surgery or radiotherapy post discussion in a multidisciplinary meeting. All patients must have received at least one cycle of immunotherapy to be eligible and must have had a baseline diagnostic MRI report indicating the presence of PNS. Patients received anti-programmed death protein 1 (PD-1) immunotherapy with either cemiplimab 350 mg/3-weekly or pembrolizumab 200 mg/3-weekly. All patients were required to have at least one additional MRI after the commencement of immunotherapy to assess PNS response.

The primary outcome was PNS response to immunotherapy as defined by our modified graded radiological criteria (Table 1). PNS is usually visualised on MRI as an abnormally thickened nerve with increased enhancement, but not exclusively. Some PNS is visualised as a thickened or mass-like nerve with minimal or no enhancement. Our assessment criteria were designed to encompass these features as an adaption from criteria previously utilised by Wu et al [15]. However we chose to develop a grading system in line with RECIST 1.1 terminology. In line with RECIST 1.1 methodology, all assessments were compared to the baseline MRI. Secondly, our definition of a partial response (PR) for PNS differed from the criteria used by Wu et al. with a PR requiring just one of improvement in perineural thickening or diminished enhancement [15]. Progressive disease (PD) criteria was also broadened to encompass new sites of disease along the nerve course. Pseudoprogression was defined as per Immune-related Response Evaluation Criteria In Solid Tumors (irRECIST) but also included patients with Delayed Response as described by Lim et al [17, 24].

**Table 1:** Perineural involvement response assessment criteria

**Complete Response (CR)**	No abnormality in nerve at follow up. Previous thickening and enhancement resolved.
**Partial Response (PR)**	Perineural thickening and/or enhancement diminished but abnormality still remains at follow up compared with baseline scan.
**Stable Disease (SD)**	Perineural thickening and enhancement is stable compared with baseline scan.
**Progressive Disease (PD)**	Perineural thickening has increased with or without enhancement compared with baseline scan. Or new sites of disease are identified along the nerve course.

PNS was reported by a radiologist with extensive expertise in head and neck malignancies. The radiologist was blinded to the historical MRI reports for each case. MRI scans reported at the > 10 month timepoint underwent a second blinded review by an additional head and neck subspecialist radiologist in order to review the reproducibility of our proposed criteria. For patients with measurable disease RECIST 1.1 was recorded. Additionally, for the subset of patients with Positron Emission Tomography Response Criteria 1.0 (PERCIST 1.0) [25] measurable disease on FDG-PET/CT, these responses were also recorded. Patients without a baseline pre-immunotherapy FDG-PET/CT were excluded from PERCIST 1.0 assessments. To create better clarity of whether PNS was detectable by FDG-PET/CT, all FDG-PET/CT studies were reviewed by a dual trained radiologist/nuclear medicine specialist with extensive expertise in head and neck malignancies who was blinded to other imaging, MRI reports or clinical information but understood that the patient had PNS detected on an MRI. Secondary outcomes included the evaluation of PNS on FDG-PET/CT imaging and a correlation between RECIST 1.1 and PNS responses. The cohort reviewed included patients treated on the phase II cemiplimab trial (NCT02760498 groups 1–3 and 5), those who received compassionate access cemiplimab due to trial-ineligibility or post trial closure, and a small number who self-funded pembrolizumab.

Descriptive statistics for baseline characteristics of patients were reported. All statistical analyses were performed in R version 4.2.1 using standard and validated statistical procedures. This study was approved by the Human Research Ethics Committee of the Peter MacCallum Cancer Centre with a waiver of consent granted.

## Results

### Baseline characteristics

In total 141 cases were reviewed which comprised of 8 patients who self-funded pembrolizumab, 80 patients who received cemiplimab via a compassionate access scheme and 53 who received cemiplimab on clinical trial. There were 20 patients who met the specified inclusion criteria. The median age was 75.7 years (range 27.9–87.1) and 75% (*n* = 15) were male. The majority of patients (85%, *n* = 17) received cemiplimab. Three patients did not have RECIST 1.1 measurable disease. All patients had locally advanced disease (16 without regional nodal involvement, four with regional nodal involvement) and no patients had distant metastases at the time of immunotherapy commencement (Table 2). Median follow up from immunotherapy commencement was 18.5 months (range: 2–59). A summary of the imaging assessments performed for each patient at each timepoint can be seen in Supplementary Table 1.

**Table 2:** Baseline Characteristics

	***N* = 20**
**Median age (years) (range)**	75.7 (27.9–87.1)
**Sex (n)**	
Male	15
Female	5
**Immunotherapy received**	
Cemiplimab	17
Pembrolizumab	3
**RECIST1.1 measurable disease at time of immunotherapy commencement**	
No	3
Yes	17
**Stage of disease at time of immunotherapy commencement**	
Locally advanced disease without regional nodal involvement	16
Locally advanced disease including regional nodal involvement	4
Distant metastatic	0

### Assessment of perineural response

**Fig. 1:**
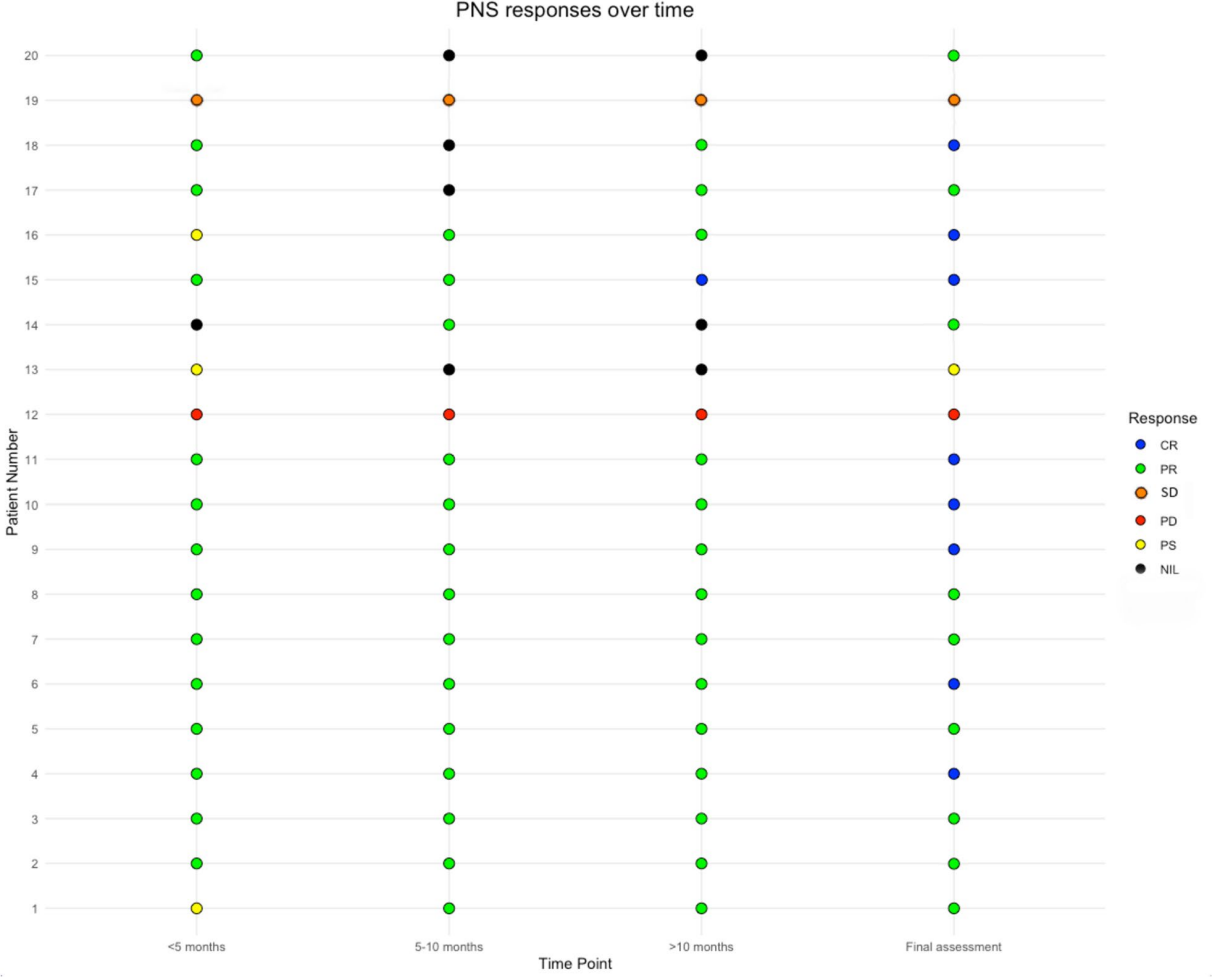
Assessment of perineural spread (PNS) over time (CR- complete response, PR – partial response, SD - stable disease, PD – progressive disease, PS – pseudoprogression, NIL – no imaging available)

**Fig. 2:**
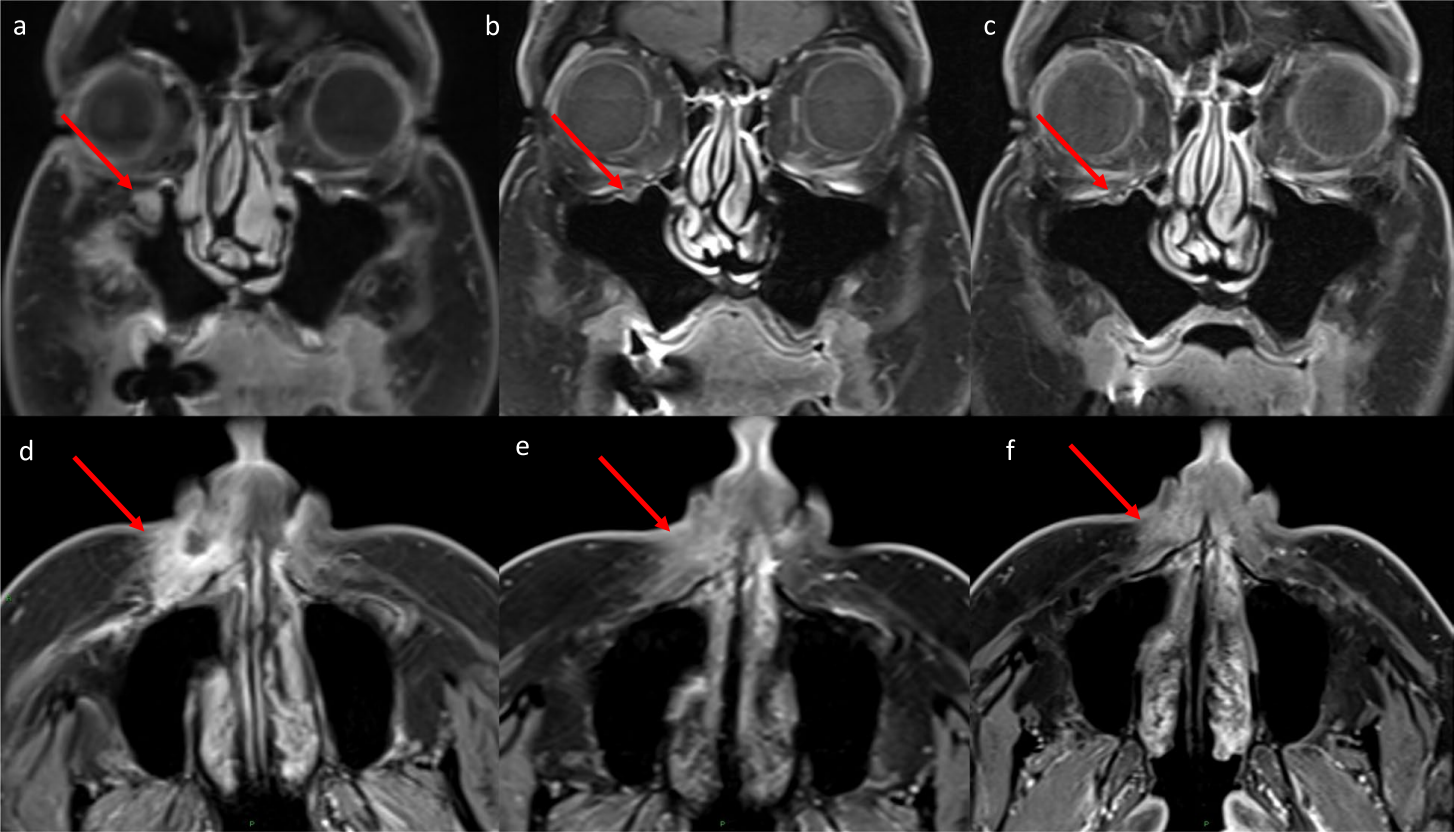
Magnetic resonance imaging (MRI) demonstrating tumour RECIST 1.1 response with concordant perineural spread (PNS) response: (**A**) All images MRI post-contrast T1 fat saturation (FS). Coronal MRI images demonstrate: (**A**) Baseline. Arrow indicates a thickened and enhancing right maxillary division trigeminal nerve (V2). **B** < 5 months image: diminished size and enhancement of the right maxillary division trigeminal nerve. **C** > 10 months image: no enhancement of the right maxillary division trigeminal nerve with normal size**.** Axial MRI images demonstrate: (**D**) Baseline. Enhancing left malar mass cutaneous squamous cell carcinoma (CSCC). **E** < 5 months, diminished enhancement and mass. **F** > 10 months (28 months), resolved enhancement and mass

A response, as measured by our Perineural Involvement Response Assessment Criteria (PNI-RAC), by 5 months occurred in 70% (n = 14) of cases. Three patients experienced pseudoprogression, one patient had a missing assessment, and one patient PD. Of the 14 patients that achieved a PR on PNI-RAC by the < 5 month scan, seven went on to experience complete resolution of their PNS by the end of study period (Fig. 1). At the end of study follow up, 8 patients had achieved a complete response (CR), 9 patients a PR, one patient stable disease (SD) and one patient experienced PD. Common features of PNS response included improvement in perineural thickening and enhancement. In the few cases where there was imperceptible enhancement, decreased thickening was the key feature. Figure 2 (A-C) demonstrates characteristic MRI changes in PNS with complete resolution over time. The 17 patients who had PNS assessments at the > 10 month interval underwent a second blinded review by an additional head and neck sub-specialist radiologist. These findings were highly concordant (94%, 16/17) with no change in the response rate identified at this timepoint.

### Correlation with RECIST 1.1

There was concordance in RECIST 1.1 and PNS assessments (Fig. 2). For the 14 cases with RECIST 1.1 measurable disease at the > 10 month time period (range: 10 -35 months) there were only two discordant PNS responses with one patient demonstrating a PR on PNI-RAC and SD as per RECIST 1.1 in their measurable disease. The other patient demonstrated a RECIST 1.1 CR in their measurable disease and a PNI-RAC PR. Cohen’s Kappa coefficient for RECIST 1.1 correlated to PNS response assessment at this timepoint was 0.62.

There were two patients who experienced RECIST 1.1 PD. The first patient had concordant PD responses in PNI-RAC and RECIST 1.1 assessments. The other patient’s tumour had a mixed response to treatment. His initial cutaneous disease involved the skin of the right zygomatic arch with infiltration of the parotid, right auriculotemporal nerve and a pre-auricular lymph node. Treatment achieved a PR on PNI-RAC, CR in disease at the right zygoma, but discordantly progressive disease at the pre-auricular site.

### PNS assessments on FDG-PET/CT

**Fig. 3:**
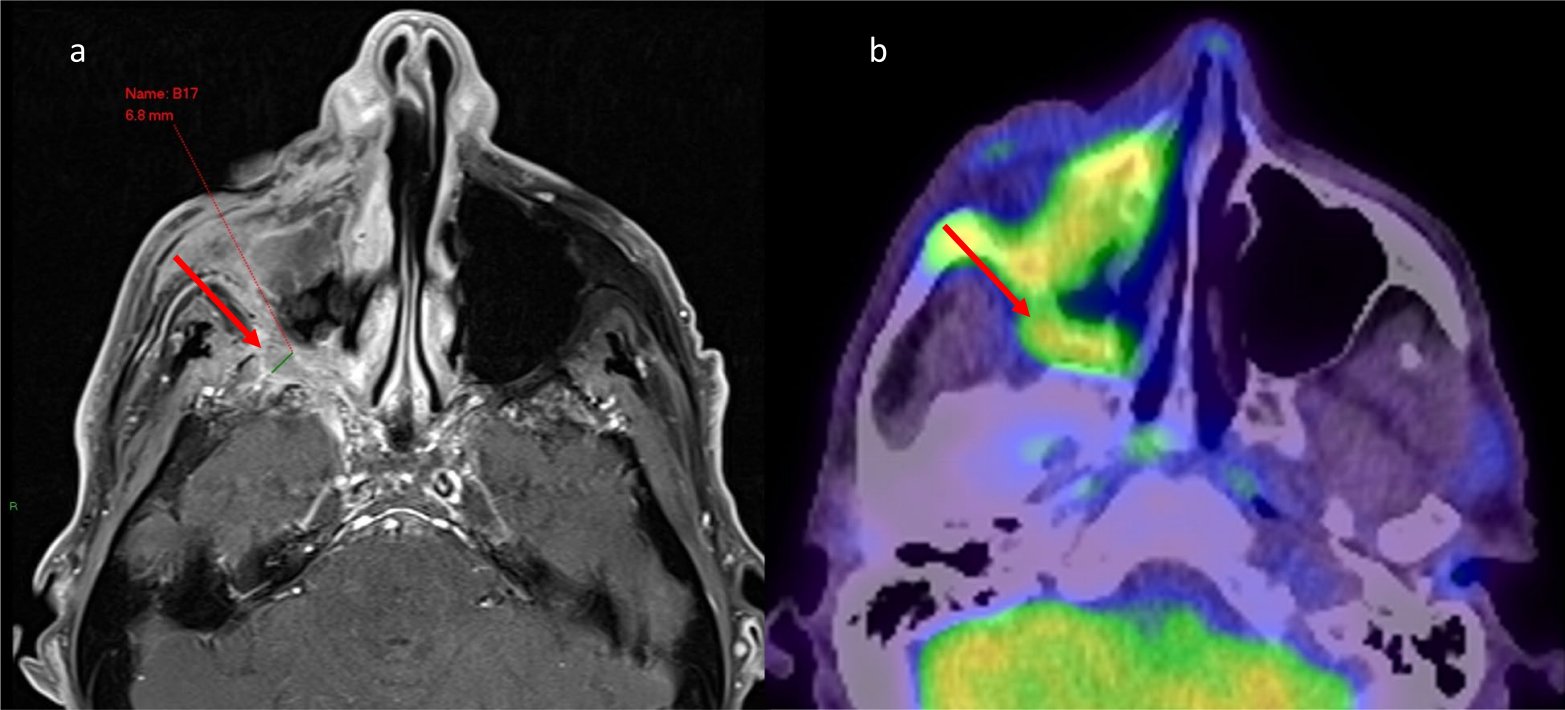
Perineural spread (PNS) demonstrated on fluorodeoxyglucose-positron emission tomography computer tomography (FDG-PET/CT), concordant with magnetic resonance imaging (MRI): Large right facial cutaneous squamous cell carcinoma (CSCC) with extensive perineural spread. **A** Axial post-contrast T1 fat saturation (FS) MRI demonstrates thickening of the right infraorbital nerve, with thickness 6-7 mm, in the right pterygopalatine fossa, right inferior orbital fissure and right infraorbital canal. This is concordant with (**B**) curvilinear FDG avidity on FDG-PET/CT in the right pterygopalatine fossa, right inferior orbital fissure and right infraorbital canal

There were 14 patients with PERCIST 1.0 measurable disease. None of the 14 patients’ FDG-PET/CT original reports confirmed the presence of PNS. On blinded review of the 14 cases, five cases were highlighted as potential PNS on FDG-PET/CT. These five cases were then reviewed for site of known PNS on MRI by the radiologist. Of the five cases only one case had RECIST 1.1 non-measurable disease, that is isolated PNS. Two of the five were large RECIST 1.1 measurable masses in the infratemporal fossa with areas of necrosis and tumour extending beyond the PNS, with the PNS expanding into the pterygopalatine fossa. Of the remaining three cases, one case was RECIST 1.1 measurable mass-like tumour encasing PNS in the orbit. One case was RECIST 1.1 measurable PNS in the posterior aspect of Meckel’s cave. The last case was MRI-isolated PNS in the V2 distribution (Fig. 3). This consisted of a large right facial CSCC with extensive PNS (PNS thickness 6-7 mm on MRI) with concordant curvilinear FDG avidity on PET in the right pterygopalatine fossa, inferior orbital fissure and infraorbital canal.

**Fig. 4:**
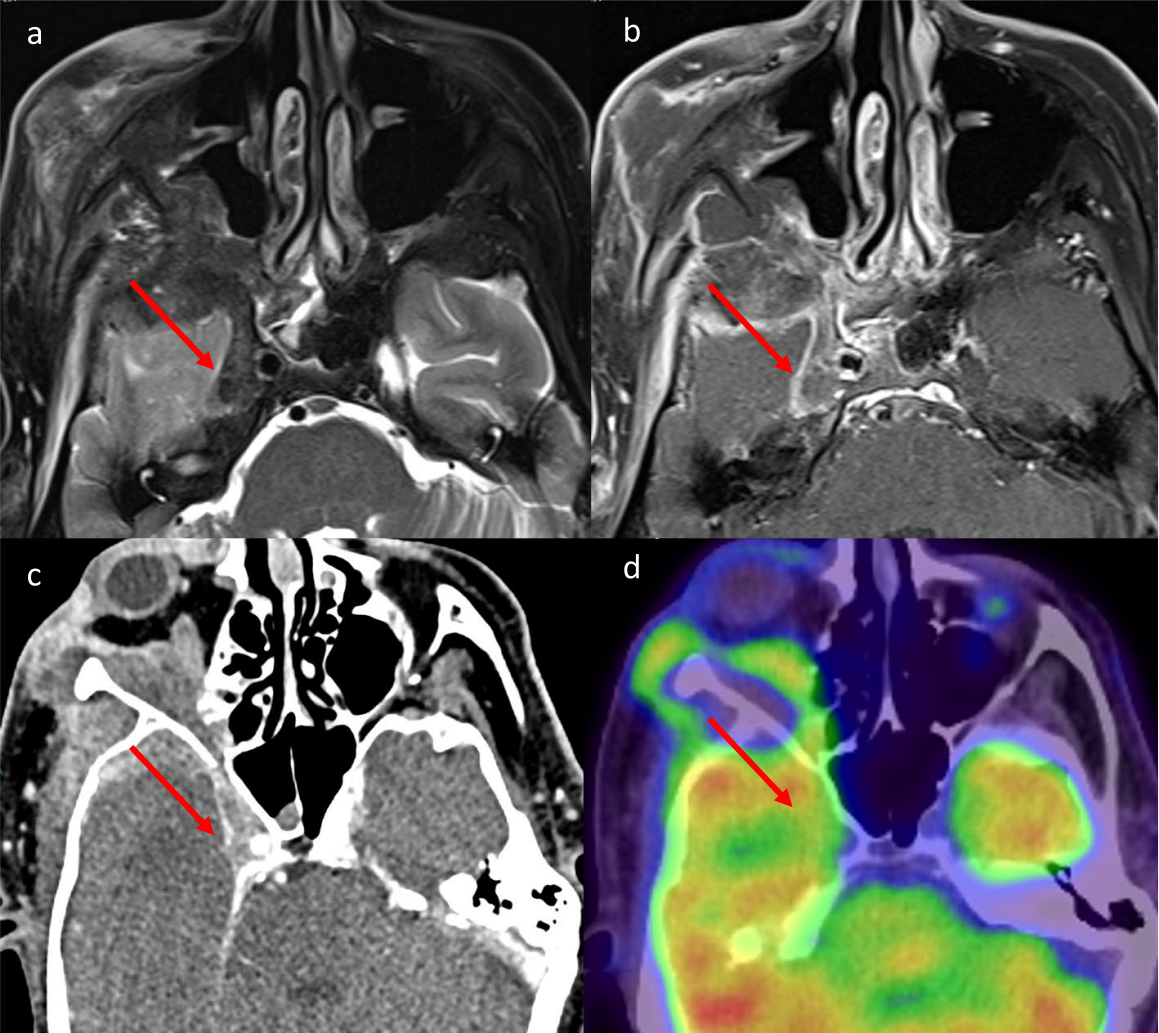
Extensive perineural spread (PNS) not detected by fluorodeoxyglucose-positron emission tomography computer tomography (FDG-PET/CT). **A** Magnetic resonance imaging (MRI) T2 axial demonstrates extensive right trigeminal nerve PNS in Meckel’s cave. **B** MRI T1 axial post-contrast fat saturation (FS) demonstrates extensive right trigeminal nerve PNS in Meckel’s cave as a mass with minimal enhancement. **C** Computer tomography (CT) post-contrast demonstrates mass like extensive right trigeminal nerve PNS in the Meckel's cave. **D** FDG-PET/CT, extensive right trigeminal nerve PNS not detected on FDG-PET/CT either obscured by FDG avidity in the adjacent brain or minimal FDG avidity in the PNS

**Fig. 5:**
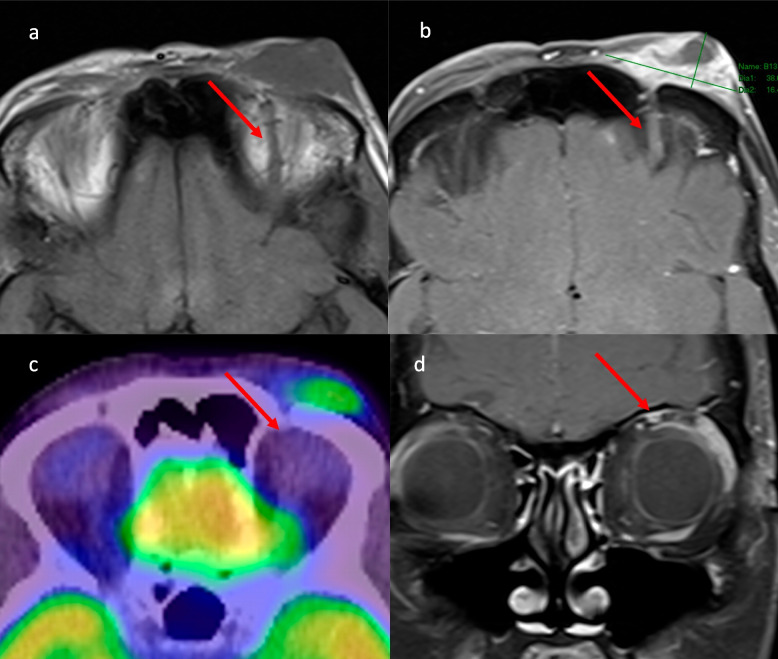
Perineural spread (PNS) in left V1, not detected by fluorodeoxyglucose-positron emission tomography computer tomography (FDG-PET/CT). Magnetic resonance imaging (MRI): (**A**) Axial T1, thickened left supraorbital nerve PNS. **B** MRI Axial T1 post-contrast fat saturation (FS), thickened left supraorbital nerve PNS with mild enhancement. **C** FDG-PET/CT axial image at the left superior orbital canal, no avidity in the PNS. PNS not detected on FDG-PET/CT. **D** MRI Coronal T1 FS post-contrast, demonstrates thickened enhancing left supraorbital nerve PNS

For the remaining 9/14 cases with confirmed PNS on MRI, FDG-PET/CT did not raise suspicion of PNS. The three main reasons for this were that the FDG avidity in the adjacent brain obscured the PNS, the PNS did not demonstrate FDG avidity, or the PNS was below the resolution of FDG PET/CT. Figure 4 demonstrates extensive PNS into the right trigeminal nerve within Meckel's cave on MRI and CT that may be identified as mass-like extension but is not detected on FDG-PET/CT. The case demonstrated in Fig. 5 shows PNS in the left supraorbital nerve that was below the resolution of FDG-PET/CT.

### Pseudoprogression

Three patients (15%) in this cohort experienced pseudoprogression.

The first patient was a 65-year-old female with a large left supraorbital CSCC who presented with numbness in the ophthalmic division of the trigeminal nerve. Post two cycles of immunotherapy her RECIST 1.1 disease and PNS had clinically and radiologically progressed but prior to surgery the supraorbital tumor auto-amputated with histopathology of the lesion demonstrating a complete pathological response.

The second patient had a CSCC over the maxilla involving the infraorbital nerve. After four cycles of immunotherapy their disease was rapidly enlarging and concerning for progression. After multidisciplinary discussion they proceeded to right maxillectomy, partial rhinectomy, orbital exenteration, neck dissection and free flap reconstruction. Histopathological review demonstrated features of pseudoprogression including extensive residual keratin with only a small area of residual CSCC in soft tissue deep to the subcutis.

**Fig. 6:**
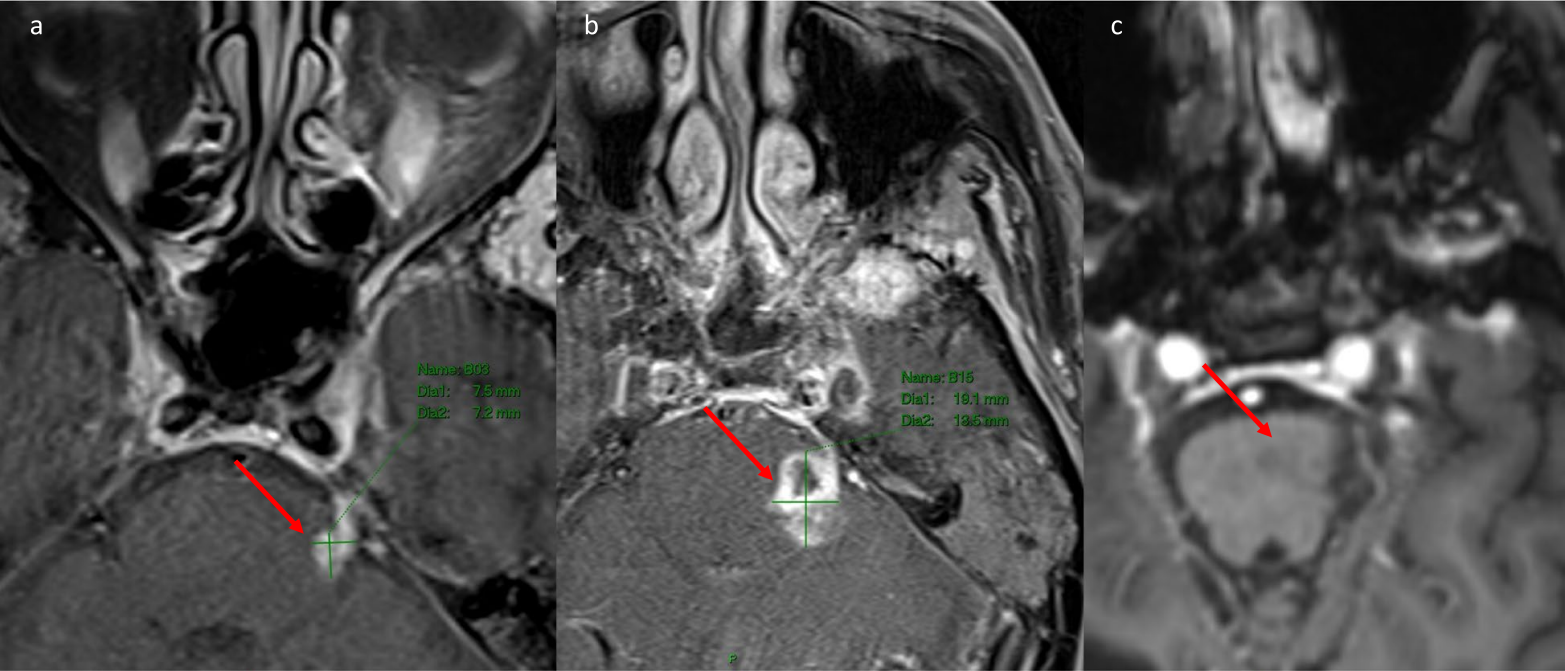
Pseudoprogression demonstrated on magnetic resonance imaging (MRI). MRI axial T1 post-contrast fat saturation (FS) axial demonstrates: (**A**) baseline perineural spread from cutaneous squamous cell carcinoma (CSCC) with mass enhancement at left pons and cisternal portion of the left trigeminal nerve. **B** < 5 months, on immunotherapy, enlarged mass-like change in the left trigeminal nerve with increased enhancement at the left pons and left Meckel’s cave. **C** > 10 months, (26 months) post immunotherapy, the left pons mass has resolved and the thickening and enhancement of the left trigeminal nerve has resolved

The third patient was an 81-year-old male with an advanced left supraorbital CSCC with perineural disease affecting the left trigeminal nerve as far as the cisternal segment. After two cycles of immunotherapy he developed ataxia and was admitted post a fall with imaging demonstrating progression of his disease with left trigeminal nerve enhancement extending into the cerebellar peduncle and pons. Pseudoprogression was suspected in this case and immunotherapy was continued. Subsequent MRI confirmed resolution of perineural changes (Fig. 6 A-C).

## Discussion

Using our modified PNS grading criteria we were able to follow PNS changes in this cohort of patients across three timepoints. Responses were graded against baseline as increased or decreased size and increased or decreased enhancement of the nerve by visual perception. PNS responses were also found to correlate with the overall RECIST 1.1 assessments (Cohen’s Kappa coefficient 0.62 at > 10 month time point). At this timepoint we were also able to demonstrate concordance (94%, 16/17) in PNI-RAC reporting by two blinded expert radiologists. This highlights that PNS responses may be observed and categorized and should have consideration of clinical value in prospective clinical trials.

There is limited retrospective data reporting on PNS responses in CSCC. The retrospective review by Wu et al. included patients treated with immunotherapy for CSCC with clinical PNI and found that 82% (9/11) had evidence of radiological perineural disease control on imaging post treatment. Of interest, they also reported only one case of complete resolution in PNS, hypothesizing that post treatment the appearance of affected nerves seemed unlikely to return to normal. Our study also demonstrated high upfront response rates in PNS (70%, 14/20) however in contrast to Wu’s report we found that 8 patients achieved complete resolution in their PNS by the end of study follow up. This may partly relate to a longer median follow up of 18.5 months in our study (compared to 13.1 months) allowing for more of these changes to occur over time. However it is also important to note when comparing these findings that there is no agreed approach on reporting PNS response and whilst similar, different assessment methods were used.

Given the increased utilisation of FDG-PET/CT as an imaging modality in CSCC, it is important to understand FDG-PET/CT’s limitations in PNS. In this study FDG-PET/CT was not able to detect the majority of radiologically detected PNS by MRI. This is not unexpected owing to the resolution limitations of FDG-PET/CT, the sometimes-limited FDG uptake, the often small volume of mass-like tissue of PNS in named nerves and the anatomical location of PNS at the base of skull directly adjacent to the brain which has physiologically intense FDG avidity, obscuring any possible PNS avidity. Armed with the anatomical knowledge of where named nerves are located, when FDG avidity is demonstrated in a curvilinear pattern at nerve locations or at anatomical bone sites widened by PNS, such as the pterygopalatine fossa, it is thought to be highly suggestive of perineural involvement [26] and in our series with expert imaging review, three patients were identified with these features. PNS disease on FDG-PET/CT is also likely to be visualized where mass-like change with measurable disease engulfs the nerve. However, PNS cases with extensive mass-like disease may still be obscured by physiologic brain FDG avidity or may not demonstrate FDG avidity. In the case demonstrated in Fig. 4, extensive PNS was identified as mass like extension in the right trigeminal nerve within Meckel's cave on MRI and CT, but was not visible on FDG-PET/CT. PNS may also be below FDG-PET/CT resolution such as shown in Fig. 5, where PNS in the left supraorbital nerve was not detected on FDG-PET/CT. Whilst other studies have highlighted FDG-PET/CT effectiveness in raising suspicion of PNS for sites such as uptake along V2 trigeminal nerve, the medial surface of the mandible or Meckel’s cave [27], it is important to emphasize two essential points: a negative FDG-PET/CT is not sufficient to exclude PNS and MRI is the imaging standard of reference for detection of PNS [28, 29]. In clinical practice when FDG-PET/CT raises the suspicion of PNS, confirmation of PNS on MRI may be required.

There is inconsistent reporting of pseudoprogression rates with immunotherapy across different tumour types in the literature. However, it is generally thought to occur in less than 10% of cases [24, 30]. In our series of three patients, all cases demonstrated increased enhancement and increased size of both their PNS and RECIST 1.1 measurable disease as an early response to immunotherapy (< 5 months time period) suggesting progression. In the first patient, auto-amputation did not enable further tumour mass evaluation however the PNS became a CR. In the second patient, orbital exenteration was undertaken because the rapid increase in tumour size was thought to be progression, yet the histopathology confirmed a major pathological response supporting pseudoprogression. In the third patient, the increased size and enhancement of the mass and PNS between baseline and < 5 months subsequently diminished in the 5–10 month interval, only revealing itself as pseudoprogression by continuing immunotherapy and monitoring after the enlargement in the < 5 month time interval. In our pseudoprogression cases the changes in PNS were concordant with RECIST 1.1 and indistinguishable from progression on imaging, highlighting the importance of recognizing this phenomenon. These cases suggest continued immunotherapy with clinical and radiological review in the early period post commencement of immunotherapy is important to determine the difference between progression and pseudoprogression. Our small series also highlights the need for better diagnostic and assessment approaches to pseudoprogression and reinforces the need for specialist multidisciplinary support in the management of these patients.

Whilst this study, to the best of our knowledge, represents the largest advanced CSCC cohort with PNS assessments post immunotherapy to date, it is limited by its relatively small sample size (*n* = 20). It is also limited by its retrospective nature with no uniformity in choice of imaging reassessments, nor in disease assessment intervals. Further prospective studies are needed to validate a PNS response assessment method in advanced CSCC.

## Conclusions

In this study we reported on a real-world cohort of 20 patients with advanced CSCC who had evidence of PNS on baseline MRI prior to treatment with immunotherapy. Using our modified graded response assessment criteria we demonstrate high response rates in PNS to immunotherapy as well as resolution of PNS changes on MRI over time in 8 patients. Additionally we demonstrate a correlation between PNS and RECIST 1.1 responses and highlight the potential for PNS responses to be followed and measured using specific criteria encompassing nerve enhancement and thickness.

### Supplementary Information

**Additional file 1.** Response assessments for the entire cohort as per perineural spread (PNS), RECIST1.1 and PERCIST1.0 criteria.

## Data Availability

The datasets generated during and/or analysed during the current study are available from the corresponding author on reasonable request.

## Abbreviations

CSCC: Cutaneous squamous cell carcinoma
PNS: Perineural spread
RECIST 1.1: Response Evaluation Criteria in Solid Tumors version 1.1
MRI: Magnetic resonance imaging
FDG-PET/CT: Fluorodeoxyglucose-positron emission tomography/computed-tomography
PR: Partial response
PD: Progressive disease
irRECIST: Immune-related Response Evaluation Criteria In Solid Tumors
PERCIST 1.0: Positron Emission Tomography Response Criteria 1.0
CR: Complete response
SD: Stable disease
